# Lung Cancer Signature Biomarkers: tissue specific semantic similarity based clustering of Digital Differential Display (DDD) data

**DOI:** 10.1186/1756-0500-5-617

**Published:** 2012-11-02

**Authors:** Mousami Srivastava, Pankaj Khurana, Ragumani Sugadev

**Affiliations:** 1 Bioinformatics Group, Defence Institute of Physiology and Allied Sciences, Lucknow Road, Timarpur, Delhi-110054, India

**Keywords:** Digital Differential Display (DDD), Lung tissue cancer, Semantic similarity, biomarker, Clustering analysis, Multiple bootstrap

## Abstract

**Background:**

The tissue-specific Unigene Sets derived from more than one million expressed sequence tags (ESTs) in the NCBI, GenBank database offers a platform for identifying significantly and differentially expressed tissue-specific genes by *in-silico* methods. Digital differential display (DDD) rapidly creates transcription profiles based on EST comparisons and numerically calculates, as a fraction of the pool of ESTs, the relative sequence abundance of known and novel genes. However, the process of identifying the most likely tissue for a specific disease in which to search for candidate genes from the pool of differentially expressed genes remains difficult. Therefore, we have used ‘Gene Ontology semantic similarity score’ to measure the GO similarity between gene products of lung tissue-specific candidate genes from control (normal) and disease (cancer) sets. This semantic similarity score matrix based on hierarchical clustering represents in the form of a dendrogram. The dendrogram cluster stability was assessed by multiple bootstrapping. Multiple bootstrapping also computes a p-value for each cluster and corrects the bias of the bootstrap probability.

**Results:**

Subsequent hierarchical clustering by the multiple bootstrapping method (α = 0.95) identified seven clusters. The comparative, as well as subtractive, approach revealed a set of 38 biomarkers comprising four distinct lung cancer signature biomarker clusters (panel 1–4). Further gene enrichment analysis of the four panels revealed that each panel represents a set of lung cancer linked metastasis diagnostic biomarkers (panel 1), chemotherapy/drug resistance biomarkers (panel 2), hypoxia regulated biomarkers (panel 3) and lung extra cellular matrix biomarkers (panel 4).

**Conclusions:**

Expression analysis reveals that hypoxia induced lung cancer related biomarkers (panel 3), HIF and its modulating proteins (TGM2, CSNK1A1, CTNNA1, NAMPT/Visfatin, TNFRSF1A, ETS1, SRC-1, FN1, APLP2, DMBT1/SAG, AIB1 and AZIN1) are significantly down regulated. All down regulated genes in this panel were highly up regulated in most other types of cancers. These panels of proteins may represent signature biomarkers for lung cancer and will aid in lung cancer diagnosis and disease monitoring as well as in the prediction of responses to therapeutics.

## Background

Gene expression analysis in the post genomic era through high throughput genomic studies led to identification of enormous candidate genes related to pathophysiological conditions or altered signal transduction. One such freely available high throughput database is ‘Unigene’ (http://www.ncbi.nlm.nih.gov/Unigene/). The Unigene libraries of interest with varying treatment conditions can be digitally ‘pooled’ and compared to control vs. treatment using Digital Differential Display (DDD). It enables the identification of numerical differences in transcript frequency between the individual or pooled Unigene libraries from the various treatment conditions and multiple cDNA libraries. The frequency of each differentially expressed transcripts and their fold change from the pooled libraries have been calculated using Fisher Exact Test. The prioritisation of DDD identification from differentially expressed candidate genes strictly used relative change in the frequency value and its fold change. Apart from DDD, many web tools are freely available to prioritise candidate genes based on the relative change in gene expression profile [1,2]. The prioritisation of each tool differs due to their different computational approaches [3]. But the process of identifying the most likely tissue specific disease candidate genes from the pool of differentially expressed genes remained difficult [1].

Recent advances in the systems biology have shown promising results in the elucidation of potential biomarkers of phenotype and clinical relevance, particularly in cancer research sphere [4-6]. These studies were performed using the predictive integration of gene expression data. Different predictive integration strategies have been developed and were used to study the biological information from public repositories [4-8]. Amongst such strategies, gene products that are biologically and functionally related would maintain similarity, both in their expression profiles and in the Gene Ontology (GO) annotation [9]. The integration of gene expression data and standardised descriptions of the biological function of gene products were used for the search of candidate prognostic biomarkers and therapeutic targets [10-12]. These studies demonstrated that the measure of functional similarity based GO annotations between query genes and the genes of interest can be applied as a complementary predictive feature to characterise gene expression profile. So, we have applied this integrative computational approach to characterise a tissue specific biological data from DDD.

We hypothesised that tissue specific differentially expressed genes can be functionally characterised using their GO semantic similarity score with normal tissue specific genes (query genes). The query genes, in this study, were normal lung tissue specific genes from the Tissue-Specific Genes Database (TiSGeD). The genes of interest were candidate lung cancer genes from DDD [13,14]. Surprisingly, this approach successfully distinguished 38 signature biomarkers for lung cancer. Thus this suggests that, in principle, this integrated methodology can offer a complementary predictive capability for detecting tissue specific signature biomarkers from the tissue specific differentially expressed data. These tissue specific signature biomarkers may be candidate prognostic biomarkers and therapeutic targets for lung cancer.

## Methods

### Selection of Human Lung Tissue specific query genes

The normal lung tissue specific genes were collected from TiSGeD (Tissue-specific gene database; http://bioinf.xmu.edu.cn/databases/TiSGeD/index.html). Human adult lung tissue related genes with tissue specificity measure score (SPM) ≥ 0.9 (represents high tissue specificity) were considered. The lung tissue specific “Mouse” and “developmental” genes were omitted.

### Collection of Lung Tissue specific differentially Expressed Candidate Genes using DDD

DDD comparisons were made at various tissue stages to elucidate the selective differential expression levels of human lung tissue specific genes for normal (Case 1) and cancerous (Case 2) conditions. In Case 1, the Normal lung tissues (11 tissue libraries) were considered as a ‘Reference’ samples and the remaining normal human tissues (251 tissue libraries) were ‘Query’ samples. In Case 2, the Normal lung tissues (11 tissue libraries) were considered as ‘Reference’ samples and the cancerous human lung tissues (8 tissue libraries) were ‘Query’ samples. These comparisons were designed systematically so as to identify altered Gene expression of varying treatment conditions of ‘Reference’ and ‘Query’ samples. These pair wise comparisons resulted in a relative abundance of ESTs among the contrasting cDNA libraries of digitally ‘pooled’ contracts from Unigene Database.

### GO-based similarity assessment

Org.Hs.eg.db package in R-program was used for the computation of Semantic similarity score while the GO-based similarity score was computed based on the three orthogonal gene ontologies generated for Molecular Function (MF), Cellular Component (CC) and Biological process (BP). GOSemSim of R-program was used to calculate semantic similarity between the GO terms and the gene products. In this study, GO terms derived from human annotations were used for calculations. The estimation of between-term similarity was based on the Wang semantic similarity measure [12]. Aggregation of between-term similarities was done with the highest between-term similarity approach, which selectively aggregates maximum between-gene similarity values [9]. Given a pair of gene products, gi and gj, annotated to a set of GO terms, the GO-driven similarity, SIM (gi, gj), is calculated by aggregating the maximum interest similarity values as follows:

$$\begin{array}{c} Sim\left(gi,gj\right)=\left[\sum_{1\leq i\leq m}max\left(Sim\left(\left(gi1\right)\left(gj\right)\right)\right)\right]\\ \;+\sum_{1\leq j\leq m}max\left(Sim\left(\left(gj1\right)\left(gi\right)\right)\right)/m+n \end{array}$$

where, two sets of GO terms gi = {gi1, gi2, …………., gim} & gj = {gj1, gj2, ……………, gjn} as query and reference sequence. Method max calculates the maximum semantic similarity score over given pairs of GO terms between these two sets, while average calculates the average semantic similarity score over a given pairs of GO terms. The hierarchical clustering of tissue specific, differentially expressed genes in relation with a normal lung tissue is shown in a Dendrogram. In the colour code of heat map, red represents a low semantic similarity below the median level, whereas, the green represents a high semantic similarity above the median level.

### Clustering analysis

The clustering analysis was carried out by the program pvclust [15]. It is an add-on package for a statistical software R to perform the bootstrap analysis of clustering and also to assess the uncertainty in hierarchical cluster analysis. The package calculates the approximately unbiased (AU) and bootstrap probability (BP) p-values for each cluster. Stability of the clustering was accessed at 95% probability (α = 0.95).

## Results

### DDD based prioritisation of lung cancer genes

In order to find the lung tissue specific differentially expressed genes, two Unigene pools (A and B) were constructed (See Additional file 1). For analysis, in the DDD1, we employed the UniGene pool (A) representing 39 human normal tissues excluding normal lung tissue and UniGene pool (B) representing 11 counterpart lung normal tissues were employed for analysis (Table 1). Similarly, in DDD2, UniGene pool (A) representing 8 human lung tumours and UniGene pool (B) representing 11 counterpart lung normal tissues were employed (Table 1). The fold change of normal lung (DDD1) and lung carcinoma candidate genes (DDD2) were calculated based on transcript frequency values. The candidate genes with an expression of at least 2-fold difference were taken into analysis. In DDD1, amongst the total of 519 differentially expressed genes 268 genes were up-regulated (≥2-fold) and 234 genes were down-regulated (≥2-fold). In DDD2, amongst the total of 203 differentially expressed candidate genes, 147 genes (≥2-fold) including 33 unknown were up-regulated (≥2-fold) and 55 genes were down-regulated (≥2-fold). Comparison of DDD1 with DDD2 has revealed that in total 76 genes from DDD1 were differentially expressed in DDD2 (See Additional file 2). From the literature survey, amongst the 76 genes, 18 of them were found to be commonly expressed in all types of cancerous conditions (See Additional file 3) [16]. Excluding these 18 from the 76, the remaining 58 genes were predicted as the lung tissue specific tumour genes (See Additional file 2). The molecular functions of these 58 genes were found to be involved in broad range of cellular functions with majority of the genes playing many different roles like structural, extracellular and intracellular functions. This subtractive approach eliminated most of the commonly expressing genes; for example, housekeeping genes. This approach has also helped to eliminate genes expressing in more than 10 cancerous conditions (See Additional file 4).

**Table 1:** Different tissue specific Unigene libraries employed in DDD

**S. No. **	**Tissue types**	**No. of EST libraries**	**dbEST identification number**	**Respective Number of ESTs**
1	Adipose	2	10983, 16445	8299, 1646
2	Adrenal gland	4	6791, 6792, 16377, 18302	4582, 1425, 2756, 10026
3	Tongue	4	12982, 18362, 18389, 18479	1116, 7730, 23564, 7420
4	Bladder	1	18307	8220
5	Blood	6	7037, 7038, 8975, 11923, 6824, 9724	1524, 1721, 4215, 6553, 7241, 9352
6	Bone	3	1124, 821, 16433	6209, 1337, 2615
7	Bone marrow	8	6975, 6976, 15949, 15950, 16412, 931, 10409, 10410	2459, 3424, 1379, 2707, 3623, 5336, 1798, 5231
8	Brain	36	1749, 16376, 16390, 18317, 18318, 18352, 18353, 18415, 18466, 14591, 14592, 186, 17380, 742, 16380, 18310, 18311, 16382, 16383, 18322, 1918, 5655, 6811, 6812, 8570, 19377, 14298, 13711, 13053, 536, 7209, 16384, 18319	1600, 1361, 13592, 2579, 15152, 43177, 24735, 23791, 6112, 31569, 15839, 1461, 1334, 18126, 5838, 4298, 3364, 41751, 40272, 2802, 3192, 16612, 7369, 8197, 2790, 3145, 19115, 44785, 1609, 7033, 3931, 4897, 5194, 2565, 25752
9	Eye	26	13915, 17747, 7316, 7315, 10273, 10287, 13901, 19465, 10274, 10281, 10280, 10288, 10279, 10284, 19471, 10285, 10286, 12093, 302, 303, 433, 10966, 10282, 10283, 16572, 13902	3739, 4253, 1836, 1294, 4005, 3543, 2785, 1334, 1595, 1479, 1115, 6010, 1469, 6719, 3043, 8344, 1199, 7816, 9190, 1732, 2174, 4531, 6279, 1185, 6097, 2946
10	Heart	8	15951, 16399, 18354, 18410, 16421, 18503, 16379, 16381	5307, 3284, 2667, 8670, 4000, 8502, 7220, 4698
11	Stomach	12	10299, 10301, 10302, 10305, 10306, 10310, 10311, 10324, 10325, 18488, 18529, 16432	1793, 2409, 1453, 2790, 1692, 1984, 6125, 5913, 1422, 8604, 2574, 3137
12	Testis	5	1752, 16441, 18476, 18517, 19376	6624, 2983, 46964, 44057, 40315
13	Thalamus	3	16437, 18348, 18349	3154, 29651, 23010
14	Thymus	6	16440, 18518, 18519, 18520, 13049, 18375	2365, 1044, 31967, 37541, 3477, 15983
15	Thyroid	3	889, 16408, 7004	1357, 4827, 3342
16	Breast	4	894, 895, 18305, 18475	1786, 6346, 2538, 8256
17	Cartilage	2	8936, 8940	4310, 3858
18	Cervix	2	18425, 18506	2674, 2619
19	Ear	2	371, 18222	12666, 3396
20	Intestine	13	840, 841, 842, 882, 16385, 18350, 18489, 16387, 16400, 17427, 18473, 16425, 18486	1499, 1704, 1759, 11996, 1817, 8191, 2619, 1351, 5536, 8199, 16855, 3403, 2545
21	Epidermis	4	20865, 21612, 7269, 21098	1627, 13186, 10681, 1135
22	Lung	11	10395, 16406, 16413, 16438, 18355, 18363, 18521, 10398, 11912, 18522, 18537	12545, 2448, 6839, 3327, 2565, 16156, 2677, 11510, 15695, 32590, 19278
23	Liver	11	1365, 12531, 12532, 12535, 12549, 12550, 13859, 16392, 18416, 18525, 18893	2302, 1607, 2315, 1136, 2425, 1561, 7537, 6856, 6550, 8424, 31921,
24	Mammary gland	3	6982, 16420, 16436	4561, 3502, 3371
25	Lymph	8	2709, 2710, 2711, 3718, 3719, 3720, 10312, 8613	3434, 3963, 1000, 9867, 1556, 1828, 7590, 1949
26	Teeth	1	12639	1576
27	Medulla	1	9725	9919
28	Muscle	4	530, 16391, 45, 18501	4271, 2154, 2485, 8276
29	Ovary	7	887, 6998, 18421, 18527, 5444, 12637, 12638	2341, 3678, 2300, 2543, 10294, 1050, 1031
30	Nose	2	358, 13908	1702, 24528
31	Placenta	14	13037, 16442, 507, 740, 6999, 10403, 10404, 10405, 10424, 10425, 16422, 17682, 18468, 18484, 13000	11885, 3501, 1370, 1172, 20941, 1862, 1166, 4260, 4517, 1271, 1529, 1032, 16852, 15843, 7344
32	Pancreas	6	16423, 422, 16960, 8840, 3884, 9821	4308, 1799, 13791, 60665, 1234, 17183
33	Prostate	8	888, 17392, 18469, 19880, 16424, 924, 928	1014, 1283, 16483, 41945, 2355, 7609, 1114, 1051
34	Uterus	6	1753, 16443, 18523, 18531, 18544, 12528	1645, 5121, 30124, 2486, 19168, 3510
35	Spleen	2	16431, 18474	2717, 33972
36	Salivary gland	1	16430	2336
37	Kidney	7	16393, 16395, 16410, 18374, 18377, 18524, 16429	5486, 5150, 3565, 17079, 2561, 15730, 1170
37	Pituitary	4	6828, 6829, 13019, 13737	3481, 1444, 7327, 1677
38	immune cells	6	1317, 17555, 17556, 8892, 12072, 12798	11447, 15272, 13685, 6112, 9223, 4548
39	Hair	4	21096, 21100, 21099, 21101	1269, 1288, 1298, 1543
40	Alimentary canal	2	18496, 18418	8371, 2586
41	Cancerous lung	8	1533, 10419, 537, 914, 14132, 14133, 14134, 14135	1377, 4401, 4173, 1850, 4365, 2121, 2847, 1560

Differentially expressed normal lung tissue specific genes (DDD1) were identified from UniGene libraries representing 39 human normal tissues (251 libraries, 1903301 ESTs, S. No. 1–21, 23–40) and counterpart normal lung tissue (11 libraries, 125630 ESTs, S. No. 22 only). Differentially expressed lung tissue specific genes (DDD2) were identified from UniGene libraries representing lung cancer libraries (8 libraries, 22694 ESTs, S. No. 41 only) and counterpart normal lung tissue (11 libraries, 125630 ESTs, S. No. 22 only).

### Prediction of Lung tissue specific tumour genes by Semantic similarity score based clustering

**Figure 1:**
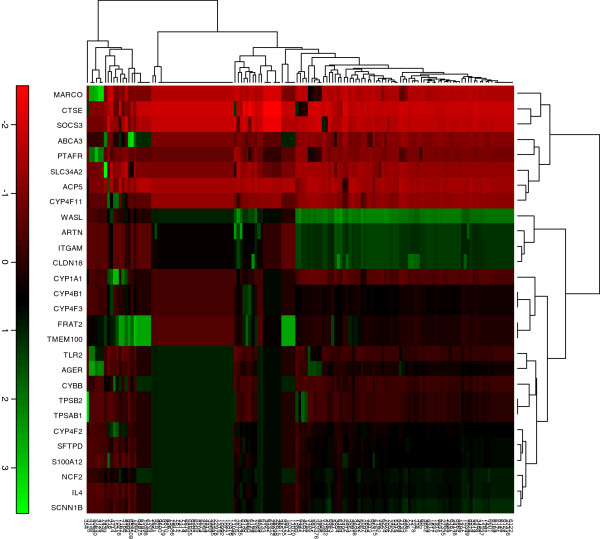
**Go semantic similarity score between the set of normal lung tissue specific genes from TiSGeD (28-horizontal, x-axis) and the differentially expressed lung cancer genes from DDD2 (145-vertical, y-axis).** The intensity of the color corresponds to the magnitude of the similarity. Red represents low semantic similarity below the median level whereas the green represents high semantic similarity above the median level.

**Figure 2:**
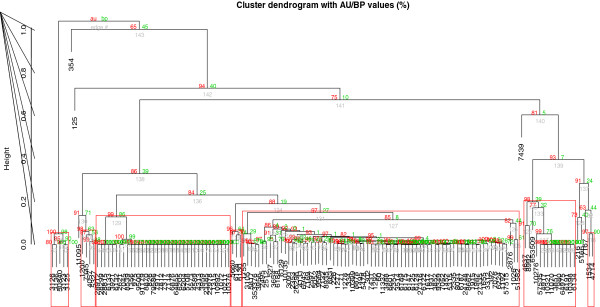
**Average correlation distances with hierarchical clustering based on GO semantic similarity score matrix calculated between normal lung tissue specific genes from TiSGed and differentially expressed lung cancer gene from DDD2.** Values in red represent **AU** (Approximately unbiased) *p*-value and green represents **BP** (Bootstrap probability) Clusters with **AU** larger than 95% are highlighted by red rectangle boxes. AU *p*-value, which is computed by multiscale bootstrap resampling, is a better approximation to unbiased *p*-value than **BP** value computer by normal bootstrap resampling.

To identify lung tissue specific clusters from the 202 genes from DDD2 cancerous condition, firstly they were subjected to similarity clustering analysis using the 47 lung tissue specific genes from TiSGeD (See Additional file 5). Before the semantic similarity clustering analysis, the Unigene ID were converted into Entrez ID. During this process, the 202 genes of DDD2 reduced to 145 and the 47 lung tissue specific genes of TiSGeD were reduced to 28 due to gene duplication. Using GOSemSim package, the similarity correlation matrix was constructed between the 145 predicted lung specific differentially expressed cancer genes from DDD2 and 28 genes from TiSGeD. The differential expression levels of these clustered genes were depicted in the form of a Heat Map (Figure 1). The similarity correlation matrix produced seven gene clusters at 95% confidence level, using the pvclust program (Figure 2). The clusters 1–4 have 14 genes and the clusters 5, 6 and 7 have 36, 74 and 14 genes respectively.

In the ID conversions from Unigene to Entrez, the 58 lung tissue specific tumour genes were reduced to 38 genes (Table 2). These 38 genes were matched with the 7 clusters. This38 genes formed four panels with the corresponding cluster 4, 5, 6 and 7 respectively. The panels 1–4 contained 2, 9, 21 and 6 genes respectively. This leads to identification of the lung tissue specific clusters of the normal lung tissue specific genes differentially regulated in lung cancer condition.

**Table 2:** Lung cancer signature biomarker clusters

**Clustering of lung tissue specific cancer Biomarkers**			**DDD1**			**DDD2**		
**Panel / Cluster of Biomarker**	**Type / class of Biomarker**	**Gene Symbol**	**Transcript frequency**		**Fold change**	**Transcript frequency**		**Fold change**
			**Pool A (normal lung tissues)**	**Pool B (Normal body tissues excluding lung)**		**Pool A (normal lung tissues)**	**Pool B (lung tissue specific cancers)**	
Panel 1 / Cluster 4	Lung cancer metastasis diagnostic markers	UCHL1	0	0.0002	-	0	0.0011	+
		LTF	0.0224	0.0018	+12.44	0.0224	0.0002	-112
Panel 2 / Cluster 5	Chemotherapy/ drug resistance related lung cancer biomarkers	TUBA1B	0	0.0002	-	0	0.0013	+
		RPSA	0.0001	0.0004	-4	0.0001	0.0027	+27
		RPL9	0.0002	0.0006	-3	0.0002	0.002	+10
		TMSB4X	0.0004	0.001	-2.5	0.0004	0.0016	+4
		COPB1	0.0007	0.0002	+3.5	0.0007	0	-
		API5	0.0007	0.0003	+2.3	0.0007	0	-
		NT5C2	0.0008	0.0003	+2.6	0.0008	0	-
		CPN	0.0009	0.0001	+9	0.0009	0	-
		PRKAR1A	0.0017	0.0006	+2.83	0.0017	0	-
Panel 3 / Cluster 6	Hypoxia related lung cancer biomarkers	FTL	0.0001	0.0011	-11	0.0001	0.0065	+65
		COL1A2	0.0001	0.0006	-6	0.0001	0.0023	+23
		GAPDH	0.0001	0.001	-10	0.0001	0.0011	+11
		IGKC	0.0002	0.0009	-4.5	0.0002	0.0016	+8
		ALDOA	0.0002	0.0006	-3	0.0002	0.0014	+7
		COL1A1	0.0001	0.0004	-4	0.0001	0.0009	+9
		FN1	0.0025	0.0012	+2.08	0.0025	0.0007	-3.57
		TGM2	0.0026	0.0008	+3.25	0.0026	0.0007	-3.71
		FOS	0.0015	0.0007	+2.14	0.0015	0.0002	-7.5
		CTNNA1	0.0034	0.0008	+4.25	0.0034	0.0003	-11.33
		FOSB	0.0024	0.0003	+8	0.0024	0.0002	-12
		APLP2	0.0109	0.0044	+2.47	0.0109	0.0008	-13.63
		NCOA4	0.0016	0.0005	+3.2	0.0016	0.0001	-16
		HIF1A	0.0011	0.0004	+2.75	0.0011	0	-
		AZIN1	0.001	0.0005	+2	0.001	0	-
		EHF	0.001	0.0001	+10	0.001	0	-
		TICAM2	0.001	0.0003	+3.33	0.001	0	-
		NAMPT	0.0008	0.0002	+4	0.0008	0	-
		TNFRSF1A	0.0008	0.0004	+2	0.0008	0	-
		DMBT1	0.0008	0.0001	+8	0.0008	0	-
		CSNK1A1	0.0007	0.0003	+2.33	0.0007	0	-
Panel 4 / Cluster 7	Lung cancer specific extra cellular matrix biomarkers	TFPI2	0	0.0004	-	0	0.002	+
		RPL10	0.0001	0.0007	-7	0.0001	0.0014	+14
		SFTPA1	0.0004	0	+	0.0001	0.0011	+11
		KIAA1324	0.0013	0.0002	+6.5	0.0013	0	-
		CRISP3	0.0009	0.0001	+9	0.0009	0	-
		NET1	0.0007	0.0002	+3.5	0.0007	0	-

**Note:** (−) represents down regulation and (+) represents up regulation. Except SFTPA1, remaining all the up regulated genes in normal condition observed to be down regulated in cancerous condition and vice versa.

We then analysed the functional significance of each panel as given below.

### Analysis of Cluster 4 / Panel 1

The cluster 4 had two-lung cancer related genes ubiquitin thiolesterase (UCHL1) and Lactotransferrin (LTF). In the normal lung (DDD1 data), UCHL1 was down-regulated and LTF was up-regulated (Table 2). This was reversed during the lung cancer condition where UCHL1 up-regulated and the LTF highly down-regulated (Table 2). These two proteins were found to be important in the cancer progression. UCH-L1 up-regulation promoted prostate cancer metastasis through epithelial-to-mesenchymal transition (EMT) induction and LTF expression decreased in lung prostate cancer progression [17,18]. Both of them were co-expressed in almost six different lung adenocarcinoma cell lines, as evident by mSigDB. This suggested that UCH-L1 and LTF could be novel diagnostic and therapeutic targets for lung cancer metastasis diagnostic markers.

### Analysis of Cluster 5 / Panel 2

The cluster 5 was playing the common functional role of immune response and complement activation. The down-regulated RPSA, RPL9, TMSB4X and TUBA1B in normal lung (DDD1) were significantly up-regulated in lung cancer (DDD2) (Table 1). The analysis resulted that all these up regulated genes played the role of tumour cell resistance to the anti-cancer agents. In gastric cancers, the up-regulation of RPSA/LRP contributed to drug resistance via hypoxia-inducible-factor dependent mechanism [19]. Similarly, there was a link between the TMSB4X and TUBA1B and the anti-cancer drug resistance to the drug Paclitaxel (PTX) observed in the cervical and breast/ovarian cancers respectively [20,21].

In this cluster, NT5C2, API5, CPN, PRKAR1A and COPB1 were fully down-regulated in lung cancer (Table 1). The down regulation of NT5C3 altered the tumour cell sensitivity to cytidine based anti-cancer drugs [22]. The anti-apoptosis gene API5 down-regulation linked to increase in the survival and resistance cancer cells to chemotherapy [23]. To our knowledge, the major copper carrying protein CPN (ceruloplasmin) down regulation link to chemotherapy/drug resistance is not yet studied. But increased level of copper in lewis lung carcinoma cells were related with the development of multi drug resistance [24]. The PRKAR1A down-regulation also linked to multidrug-resistant (MDR) in colon carcinoma cells [25]. The COPB1 was an essential component for the coatomer formation [26]. These coatomers were involved in the drug trafficking pathways and endocytic drug delivery [27]. So, it was expected that the down-regulation of COPB1 might have a role in the chemotherapy which needs to be taken up and studied. We are surprised to find that all these results suggest that the cluster 5 functionally represents a panel of chemotherapy/drug resistance related lung cancer biomarkers.

### Analysis of Cluster 6 / Panel 3

In cluster 6, the upregulated FTL (65 fold in our study) and ALDOA (7 fold in our study) were regulated by hypoxia inducible factor (HIF) during lung cancer [28-31]. The COL1A1 (23 fold in our study) and GAPDH (11 fold in our study) were regulated by hypoxia [32-34]. IGKC (8 fold in our study) up-regulated in lung cancer patients but no literature data was available for its interaction either with HIF or hypoxia [35]. The HIF, TGM2, CSNK1A1, CSNK2A1, CTNNA1, NAMPT)/Visfatin, TNFRSF1A, ETS1 and SRC-1 were down-regulated and proposed as the biomarkers for lung cancer. We found all of them to be interacting with the HIF in cancerous condition [36-45]. The down- regulated FN1 and APLP2 showed hypoxia dependent differential regulation [46-48]. The DMBT1/SAG interacted HIF-1 was a kind of feedback loop in response to hypoxia. The hypoxia induced HIF-1 to transactivate SAG and the induced SAG then promoted HIF-1alpha ubiquitination and degradation [49]. The FBJ/c-Jun/AP-1 interacted with HIF during hypoxia that controlled the transcriptional regulation of the Cyr61 gene in retinal vascular endothelial cells [50]. The role of AIB1/SRC-3/NCoA during hypoxia condition were exhibited by controlling the expression levels of HIF induced erythropoietin (EPO) gene during hypoxia [42].

However, in this cluster, the AZIN1 and TICAM2 were down-regulated and were lacking direct experimental evidence to support their regulation with HIF or hypoxia during cancer. The following literature analysis suggests their possible regulations either with HIF or hypoxia. The AZIN1 was an inhibitor for the antizyme and both were highly regulated in human cancers and antizyme induced HIF, during increased cellular redox potential [51-53]. The TICAM2 physically bridged toll like receptor-4 (TLR4) with TICAM1 and the TLR4 partially regulated by the HIF during adenocarcinoma [54,55].

All these results suggest that the cluster 6 represents the panel of either HIF or Hypoxia related lung cancer biomarkers.

### Analysis of Cluster 7 / Panel 4

In the Cluster 7, there were seven lung biomarkers, mostly encoding for lung tissue specific extra cellular matrix proteins. The epigenetic analysis using Methycancer database (http://methycancer.genomics.org.cn) revealed that amongst the seven, KIAA1324, NET1, NTN3, RPL10 and TFPI2 were epigenetically regulated through DNA methylation. In the remaining two, SFTPA1 was epigenetically regulated [56-58]. However, the experimental evidence was lacking the epigenetic related data for CRISP3. However, the Gene card database analysis of CRISP3 showed that the CRISP3 orthlogous gene C-type lectin domain family 18 member A (CLEC18A) epigenetically regulated through DNA methylation (http://www.genecards.org). All these results show that the cluster 7 represented the panel of epigenetically regulated lung cancer specific extra cellular matrix biomarkers.

## Discussion

UniGene database using the DDD tool provides us a computational approach to study and understand the lung tissue specific gene expression levels in both disease and normal conditions [59]. Studying their differential expression in disease state (lung cancer) will provide a clue about lung cancer specific candidate genes. However, the candidate identification of the DDD method is relying on the EST frequencies based fold change calculation. In DDD2, the 203 differentially expressed candidate genes (≥2-fold) ranking / prioritisation only based upon fold change did not account for the tissue specific variability of the genes in disease conditions (eg: biomarker identification). To include the tissue specific variability in DDD2 prioritisation, the normal lung tissue specific genes from DDD1 were compared. This approach eliminated most of the house keeping genes from the analysis (gene list reduced from 202 to 76). Further, we detected gene*s* expression selectively altered in the lung cancer by eliminating genes that commonly expressed differentially in more than five tumours (gene list reduced from 76 to 58) (See Additional file 2). Almost all of them have a documented role in the lung cancer (http://www.megabionet.org/bio/hlung). So, these subtractive approaches successfully increase the probability of identifying the lung cancer specific probable candidate biomarkers.

The semantic similarity scores amongst the GO terms and the subsequent hierarchical clustering were calculated using the freely available R-software for lung tissue specific candidate genes from normal and cancer conditions. The analysis of members of individual genes from each cluster revealed the functional significance of each cluster. Out of the seven clusters, our approach identified four functionally important clusters. The four clusters represented metastasis diagnostic markers, chemotherapy/drug resistance related biomarkers, and HIF or Hypoxia induced biomarkers and epigenetically regulated extra cellular matrix biomarkers for lung cancer. This suggests that, especially for lungs tissues, the semantic similarity score amongst GO terms between normal and diseases condition from the same tissue can prioritise biomarkers. But, further study is necessary to extend our hypothesis to other tissues. This subtractive approach integrated with semantic similarity score among GO terms can offer a predictive capability for detecting tissue specific signature biomarkers from the tissue specific differentially expressed data. This approach is also complementary to the network based biomarker prediction approach [60,61]. Our study is one more example of demonstrating the utility of the Digital differential expression technique.

Our study suggests that amongst the 4 panels, HIF or Hypoxia induced lung cancer biomarkers panel (panel 3) is the most important cluster. Because, in other clusters, most of the identified lung cancer biomarkers follow the same expression pattern (either up or down) in other types cancers like breast, ovarian, cervical etc. However, in our study and literature, the expression pattern of genes down regulated in cluster 6 / panel 3 is distinct from almost all types of other cancers. In panel 3, the expression pattern of the HIF and its modulating proteins are completely different when compared to most of the other types of cancers. For example, in most of the cancerous conditions the HIF level is up-regulated [62]. This up-regulation is expected in cancers due to the acute hypoxic condition exhibited during cancer. In contrast, in lung cancer, the HIF level is completely down-regulated (Table 1).

Therefore, it is evident from our study that the HIF down regulation also affect the expression level of the other HIF modulating lung cancer biomarkers. All the down-regulated genes, in this Panel 3 showed their significant up-regulation in most of many types of cancers (TGM2 [63,64], CSNK1A1 [65], CTNNA1 [66], NAMPT/Visfatin [67], TNFRSF1A [68], ETS1 [41], SRC-1 [69], FN1 [70], APLP2 [71], DMBT1/SAG [64], AIB1 [72], AZIN1 [72]). Our study further shows that this down-regulation is more than five folds when compared to the normal lungs tissue (Table 1). This fold change level suggests that this fold change seems to be more than enough to detect them in the patient sample. Therefore, this panel of down regulating HIF / hypoxia regulated lung cancer biomarker can help to distinguish lung cancer from other types of cancers.

The identified 38 signature lung cancer specific biomarkers can help to increase the sensitivity and selectivity for early diagnosis of lung cancer.

## Conclusion

We could demonstrate that our approach readily predicted lung tissue specific cancer biomarkers from digital differentially expressed lung cancer tissue specific genes. The procedure can easily adapt for the prediction of tissue specific biomarkers from the tissue specific differentially expressed genes. It is necessary to explore the extent to which the proposed approach can be integrated with the prediction of tissue specific biomarkers from tissue specific microarray datasets.

## Abbreviations

ESTs: Expression Sequence Tags; DDD: Digital Differential Display; GO: Gene Ontology; TiSGeD: Tissue-Specific Genes Database; EMT: epithelial-to-mesenchymal transition.

## Competing interests

The authors declare that they have no competing interest.

## Authors’ contributions

RS conceived the project, participated in its design, interpretation of the result and drafted the manuscript. MS design protocol and carried out statistical, computational analysis and interpretation of the result. PK contributed to statistical analysis. All authors read and approved the final manuscript.

## Supplementary Material

Additional file 1 **Table S1. DDD1-** The complete list of differentially expressed normal lung tissues (11 libraries) and other normal tissues (251 libraries) with their fold change and transcript frequency values of Pool A and B. **Table S2.** DDD2- The complete list of differentially expressed normal lung tissues (11 libraries) and lung cancer tissues (8 libraries) with their fold change and transcript frequency values of Pool A and B. **Table S3.** The DDD1complete conversion list of unigene identifier to Entrez gene id. **Table S4.** The DDD2complete conversion list of unigene identifier to Entrez gene id. Click here for file

Additional file 2 **Table S1.** The complete lists of 76 genes from DDD1 were differentially expressed in DDD2. **Table S2.** The complete list of 58 genes after removing 18 genes expressed in all types of cancers from the 76 genes. Click here for file

Additional file 3 **Table S1.** Number of unique Unigene identifiers and ≥2 fold present in DDD1 and DDD2 Figure: Three- way Venn diagram of DDD1, DDD2 and genes expressing in all types of cancers. **Table S2.** The complete list of genes and their symbols in different intersections (A to G) of Venn diagram were given. Click here for file

Additional file 4 **Table S1.** The complete list of genes expressed in all type of cancers (Chen et al., [2006]). Click here for file

Additional file 5 **Table S1.** The complete list of Adult Human Lung Tissue specific genes from TiSGeD having SPM ≥ 0.9. **Table S2.** The TiSGeD gene symbol conversion to Enterz ID. Click here for file
