# Supplementary material for: Lung Cancer Signature Biomarkers: tissue specific semantic similarity based clustering of Digital Differential Display (DDD) data
Source: BMC Res Notes. 2012 Nov 2;5:617. doi: 10.1186/1756-0500-5-617 (PMC3532198; doi:10.1186/1756-0500-5-617)
Supplement: Additional file 3 — Table S1. Number of unique Unigene identifiers and ≥2 fold present in DDD1 and DDD2 Figure: Three- way Venn diagram of DDD1, DDD2 and genes expressing in all types of cancers. Table S2. The complete list of genes and their symbols in different intersections (A to G) of Venn diagram were given. [file 1756-0500-5-617-S3.pdf]

Table 1. Number of Unigene identifiers in DDD1 and DDD2

	DDD1	DDD2
Number of Unigene Identifiers	519	203
Number of Unigenes after Fold Change ≥ 2 cutoff	502	202
*Number of Unigenes after removing gene duplication (fc ≥ 2).	472	182

*Same gene Symbol but different Unigene Identifier

List	#	
1	472	
2	182	
3	220	
1 only	373	**a**
2 only	88	**b**
3 only	161	**c**
1 & 2	76	**d + g**
2 & 3	36	**e + g**
1 & 3	41	**f + g**
1 & 2 & 3	18	**g**
1 & 2 only	58	**d**
2 & 3 only	18	**e**
1 & 3 only	23	**f**

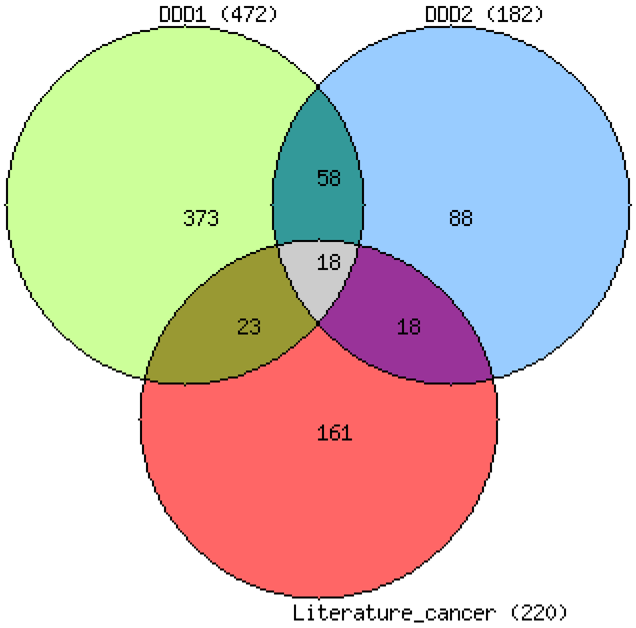

Figure. Three- way Venn diagram to compare DDD1, DDD2 and genes expressing in all types of cancers.

Table2. The complete list of genes and their symbols in different intersections (A to G) of venn diagram.

A(373)	B(88)	C(161)	D(58)	E(18)	F(23)	G(18)
ABCA13	ACPP	AARS	ADH1B	ANXA2	A2M	ACTG1
ABCD3	ADAMTS8	ACLY	ALDOA	CFL1	APOE	ALDH1A1
ABP1	AP2A1	ACTA2	API5	CTSD	CANX	EEF1A1
ACO1	ARHGAP1	ACTB	APLP2	EEF1G	CAV1	FTH1
ACRBP	ATP6V0C	ACTG2	AZIN1	HLA-A	CD55	GNB2L1
ACSL1	ATP6V0E1	ADAR	C10orf10	HSPA8	CLIC4	PPIA
ACTA1	BAT3	AHCY	CFH	LGALS1	CRYAB	RPL10
ADAM12	BCAR1	ANXA11	COL1A1	MAP1B	GPX3	RPL13A
ADH7	C1RL	AP1M1	COL1A2	MYL6	HBA2	RPL3
ADIPOQ	CAPNS1	APOD	COPB1	MYL9	HYOU1	RPL7A
AFP	CD63	APP	CP	RPL4	KRT7	RPL8
AGER	CD81	ARPC1B	CRISP3	RPL5	KRT8	RPLP0
AGT	CD99	ASS	CSNK1A1	RPL6	LDHB	RPS2
AIDA	CDKN1A	ATP5A	CTNNA1	RPL7	PTGDS	RPS20
AKAP2	CHPF	ATP5B	CYBRD1	RPS10	RPL10A	RPS3
ALB	CKAP4	B3GALT4	DMBT1	SPARCL1	RPL31	RPS3A
ALCAM	CLIC1	BAL	EHF	TIMP1	RPL37	RPS4X
ALDH18A1	COL6A2	BASP1	FN1	VIM	RPS23	UBB
ALDH1A3	COPE	BCL2L1	FOS		RPS27	
ALDOC	CSDE1	BHLHB3	FOSB		RPS6	
APLP1	CYB561	BRI3	FTL		RPS8	
APOL1	DAZ1	BSG	GAPDH		STMN1	
APOL3	DDX5	C	HIF1A		THY1	
AQP1	EIF3B	C1	HSPA1A			
AQP3	EIF3K	CALM1	IGKC			
ARPP21	EIF4G2	CCDC5	IGL@			
ATCAY	FAM129B	CCT3	KIAA1324			
ATF3	FCGRT	CCT7	KLK3			
ATP12A	FLOT1	CD14	LDLR			
ATP1A3	GABARAP	CD46	LOC100287276			
ATP5A1	GAS5	CD59	LOC100288550			
ATP6V0A1	GAS6	CDKN1C	LOC100293559			
ATP6V0E2	GDF15	CLSTN1	LTF			
B3GNT5	GPX1	CLTC	MUC7			
B4GALT1	HLA-DRB1	CNAP1	NAMPT			
BDKRB2	IER3	COL3A1	NCOA4			
BHMT	IFITM3	CORO1A	NET1			
BIRC2	IGFBP4	COX5B	NT5C2			
BPIL1	IGFBP5	COX7C	PDK4			
C10orf81	IRAK1	CRAB	PRKAR1A			
C12orf74	ITGB5	CTDSP2	PROM1			
C13orf30	KIAA1191	CYB5	PRR4			
C1orf116	KRT4	CYBB	RPL9			

C1orf88	KRT5	CYCS	RPSA			
C6orf58	LGALS3BP	DDIT4	SCFV			
CA1	LOC100289173	DDX1	SFTPA1			
CA3	LRP10	DDX24	SLC2A3			
CALCRL	LRRC41	DGCR2	TFPI2			
CALM2	MAGED1	DNMT1	TGM2			
CALR	MAT2A	DTX1	TICAM2			
CAMKK2	MMP2	DUSP1	TMSB10			
CAMKV	MRPL41	EEF2	TMSB4X			
CAPN2	NCRNA00188	EIF2S3	TNFRSF1A			
CCDC17	NCSTN	EIF3S6	TPI1			
CD200R1	NDUFB8	EIF3S8	TUBA1A			
CD302	NNMT	EIF4A1	TUBA1B			
CD4	PKM2	ENO1	TXNRD1			
CD44	PLAT	EPB41L2	UCHL1			
CD83	PLAU	FADS1				
CDH1	POFUT2	FNBP1				
CDHR3	PRDX1	FPGS				
CEACAM5	PRPF6	FXYD6				
CGA	PSAP	G6PD				
CKB	PSMD2	GAPD				
CKM	PVR	GGA2				
CKMT1B	QSOX1	GLUL				
CLCA2	RBMX	GNB2				
CLDN1	RPL14	GRF2				
CLDN12	RPL23	GRLF1				
CLDND1	RPL23A	HLA-C				
CLU	RPS11	HLA-DPA1				
CNP	RPSAP58	HLA-E				
COCH	SCARF2	HMGA1				
CORO7	SERINC1	HMGN2				
CPA1	SF3B2	HNRPA1				
CPB1	SFTPC	HSPB1				
CPD	SNRPC	HSPCB				
CPLX2	SQSTM1	HSPD1				
CRYAA	SRPR	IMPDH2				
CRYBB2	SYVN1	ITGB4				
CSF3	TAGLN	JUN				
CSH1	TCF21	K-ALPHA-1				
CSH2	TGFBI	KPNB3				
CSRP1	TMEM115	KRT18				
CTNNB1	TPM2	KRT19				
CYFIP2	TPP1	LAMR1				
CYP11A1	UBA1	LDHA				
CYP17A1	VASN	LMNA				
CYP19A1		M6PRBP1				
CYP1B1		MKI67				
CYP21A2		MSH6				

CYP2B7P1<br>CYP4B1<br>CYP4X1<br>D4S234E<br>DDR1<br>DDX3X<br>DDX3Y<br>DES<br>DLC1<br>DLG5<br>DLK1<br>DNAH12<br>DNTTIP2<br>DUOX1<br>DUSP6<br>DYDC2<br>ECT2L<br>EFCAB1<br>EHD1<br>EHD2<br>EIF5A<br>ELF1<br>ELF3<br>ELN<br>ENG<br>ENO2<br>ENO3<br>EPAS1<br>ERAP1<br>ERAP2<br>ERMN<br>ERN2<br>EXPH5<br>F11R<br>F13A1<br>F3<br>FAM107A<br>FAM154B<br>FAM46C<br>FBXL3<br>FCN3<br>FIGF<br>FLNA<br>FMO2<br>FMO5<br>FRMD4B<br>FSTL1<br>FZD6		MYH11<br>NDRG2<br>NDUFA4<br>NDUFA5<br>NONO<br>NPM1<br>NSEP1<br>NUD<br>OK/SW-<br>cl.56<br>P2<br>P40<br>PABP<br>PEA15<br>PGK1<br>PHGDH<br>PIK3CD<br>PKM<br>PORIMIN<br>PPP1R16B<br>PRKWNK1<br>PSMB6<br>RAB31<br>RAD23B<br>RAN<br>RBM3<br>RERE<br>RPL21<br>RPL27A<br>RPL38<br>RPL41<br>RPS19<br>RPS24<br>RPS5<br>SBF1<br>SC5DL<br>SCD<br>SDHD<br>SEPP1<br>SERPINA3<br>SERPINF1<br>SIAT1<br>SLC25A3<br>SLC25A6<br>SLC2A1<br>SLC3<br>SLC40A1<br>SLC7A5				

GABBR1<br>GABRP<br>GALC<br>GALNT3<br>GALNT4<br>GANAB<br>GCLC<br>GDI1<br>GFAP<br>GH1<br>GNAS<br>GNE<br>GNS<br>GP2<br>GPD1L<br>GPI<br>GPM6A<br>GPM6B<br>GPR110<br>GPRC5A<br>GPX8<br>GRM3<br>H2AFZ<br>HADHB<br>HBB<br>HBG2<br>HHAT<br>HIST1H4C<br>HMP19<br>HSPA2<br>HSPA6<br>ICAM1<br>IGHG1<br>IGHV1-18<br>IGJ<br>IRAK3<br>IRF6<br>ITGA5<br>KIF1A<br>KLK2<br>KPNA2<br>KRT1<br>KRT12<br>KRT13<br>LAMB2<br>LARS<br>LDB3<br>LDLRAD1		SMARCA4<br>SORL1<br>SPRED2<br>SPTBN1<br>SRP9<br>SRRM2<br>SYK<br>TACSTD2<br>TBC1D1<br>TIMP3<br>TKT<br>TLN1<br>TRAP1<br>TU3A<br>TUBA3<br>TUBA6<br>TUBB2<br>UBE2<br>UBE2J1<br>UBE2R2<br>USP22<br>VAMP2<br>ZNF151				

LIPF<br>LOC100287415<br>LOXL2<br>LOXL4<br>LPO<br>LRIG3<br>LRRIQ1<br>LYZ<br>MAEL<br>MAFF<br>MAG<br>MAGT1<br>MALAT1<br>MAPRE2<br>MATN1<br>MB<br>MBP<br>MCFD2<br>MET<br>MLC1<br>MLLT11<br>MOBP<br>MOV10<br>MPZL2<br>MS4A8B<br>MTF1<br>MYL2<br>MYO1C<br>NAA50<br>NAPSA<br>NCAM1<br>NDRG1<br>NDRG4<br>NEFL<br>NEFM<br>NKX2-1<br>NR4A1<br>NSF<br>NT5E<br>NUP62<br>OXR1<br>PAM<br>PAQR6<br>PDE4D<br>PEBP1<br>PEX13<br>PGA3<br>PGA4						

PHF7<br>PIGR<br>PITRM1<br>PLEKHB1<br>PLP1<br>PLS1<br>PNLIP<br>PODXL<br>POF1B<br>POMC<br>PRDX2<br>PRKAA1<br>PRKCZ<br>PRL<br>PRM2<br>PRNP<br>PRSS1<br>PRSS23<br>PSG1<br>PSG3<br>PSG4<br>PTMA<br>PTRF<br>PUM1<br>QDPR<br>RAB10<br>RBM47<br>REG1A<br>REG1B<br>RGS1<br>RHO<br>RHOA<br>RHPN2<br>RND3<br>RPH3A<br>RPL13<br>RPL18<br>RPL18A<br>RPL28<br>RPL30<br>RPL35<br>RPL36<br>RPL37A<br>RPLP1<br>RPLP2<br>RPS15<br>RPS17<br>RPS18						

RPS29<br>RPS9<br>RSAD2<br>RTN1<br>RTN3<br>SAMD12<br>SARNP<br>SCARA5<br>SCNN1A<br>SEC31A<br>SEC61A1<br>SELL<br>SEMG2<br>SERF2<br>SERP1<br>SERPINA5<br>SERPINB3<br>SERPINB4<br>SERPINH1<br>SFTA3<br>SFTPB<br>SFTPD<br>SH2D4A<br>SH3BGRL2<br>SH3GLB2<br>SLC15A2<br>SLC1A2<br>SLC1A3<br>SLC33A1<br>SLC34A2<br>SLC44A4<br>SLC5A1<br>SLC5A8<br>SLC6A14<br>SLC6A4<br>SLFN13<br>SLITRK6<br>SLK<br>SNAP25<br>SND1<br>SOD2<br>SPAG17<br>SPARC<br>SPOCK1<br>SPP1<br>STAR<br>STMN2<br>STOML3						

STXBP1<br>SUSD2<br>SV2A<br>SYT4<br>TBL1XR1<br>TC2N<br>TCF4<br>TCP11<br>TEKT1<br>TF<br>TG<br>THBS1<br>THBS3<br>TMC5<br>TMEM100<br>TNFAIP3<br>TNFRSF19<br>TNNC2<br>TNNI1<br>TPT1<br>TRIM26<br>TSPAN19<br>TSPAN5<br>TSPAN7<br>TTN<br>TUBB2B<br>TUBB4<br>UMOD<br>UNC45A<br>UTRN<br>VCP<br>VNN3<br>VTCN1<br>VWA5A<br>WARS<br>WBP2<br>WDR49<br>WDR78<br>WWP2<br>XAF1<br>YSK4<br>ZFP36L1						
